# An unusual artifact observed on screening mammography in a patient with an LVAD

**DOI:** 10.1002/acm2.14255

**Published:** 2024-01-05

**Authors:** Audrey I. Nisbet, David Ahmadian, Srinivasan Vedantham, Jing‐Tzyh Alan Chiang

**Affiliations:** ^1^ Department of Medical Imaging University of Arizona Tucson Arizona USA; ^2^ College of Medicine University of Arizona Tucson Arizona USA

**Keywords:** artifacts, breast imaging, electromagnetic interference, image quality, left ventricular assist device, mammography, tomosynthesis

## Abstract

**Purpose:**

Screening mammography and digital breast tomosynthesis consist of high‐resolution x‐ray images to identify findings that are potentially indicative of breast cancer, enabling early detection and reduction of breast cancer mortality. Imaging artifacts can occasionally occur, sometimes due to patient‐related medical devices. Because of continuous evolution of new technologies, there is potential for novel artifacts to be encountered. In this technical note, we report an unusual artifact in the screening mammogram of a patient with an Abbott HeartMate 3 left ventricular assist device (LVAD).

**Methods:**

A 72‐year‐old patient with a HeartMate 3 LVAD presented to our breast imaging facility for a standard screening exam with digital breast tomosynthesis (Selenia Dimensions, Hologic Inc., Bedford, MA) and synthetic 2D images (C‐view, Hologic Inc., Bedford, MA).

**Results:**

Linear artifacts oriented in the anteroposterior dimension demonstrating a spatial periodicity of ∼1.4 mm were seen on all left breast images, whereas concurrent right breast images did not demonstrate any artifacts. Repeat attempts using two identical digital breast tomosynthesis units demonstrated the same artifacts. No other exam at our imaging center that day demonstrated any such artifacts. Mammogram exams performed on this patient prior to her LVAD placement did not exhibit any similar artifacts.

**Conclusion:**

Findings support the patient's LVAD as the underlying source of linear artifacts observed on left breast images, particularly given the proximity of the LVAD to the left breast. With the number of patients receiving LVAD placement on the rise, as well as increasing median survival rates status post LVAD implantation, recognition of this LVAD related artifact on mammography may be important.

## 1 INTRODUCTION

Screening mammography and digital breast tomosynthesis are important tools for early detection of breast cancer.^1^, ^2^, ^3^ Current guidelines from the American College of Radiology and Society of Breast Imaging recommend annual screening mammography for all women ages 40 and older.^2^, ^4^ In the United States, over 39 million patients undergo mammographic evaluation every year,^5^ including over 60% of American women ages 40 and above.^6^ As a result of screening mammography programs, breast cancer mortality reduction has been estimated to be as high as 40%.^7^, ^8^

Mammographic exams consist of high‐resolution x‐ray images to identify findings that are potentially indicative of breast cancer.^9^, ^10^ Modern mammographic technology allows for acquisition of exams with consistently high image quality and throughput.^9^, ^11^ However, imaging artifacts can occasionally occur, and many commonly seen artifacts in mammography and digital breast tomosynthesis have been previously described and reviewed.^12^, ^13^ These well‐documented artifacts are often related to mammographic equipment hardware and software, as well as patient‐related medical devices. Because of continuous evolution of new technologies, there is potential for novel artifacts to be encountered.

A left ventricular assist device (LVAD) is a mechanical device that replaces cardiac function in patients with severe heart failure.^14^, ^15^ Initially designed to sustain cardiac function while patients are awaiting heart transplant (i.e., “bridge to transplant”), LVADs were subsequently approved as end treatment for heart failure as well (i.e., “destination therapy”).^15^, ^16^ In the United States, over 2000 patients receive an LVAD device annually,^17^, ^18^ and over 27 000 patients have benefitted from LVAD placement since 2012.^16^

In this technical note, we report an unusual artifact in the screening mammogram exam of a patient with an Abbott HeartMate 3 LVAD.

## 2 METHODS

A 72‐year‐old patient with a HeartMate 3 LVAD presented to our breast imaging facility for a standard screening exam. Bilateral craniocaudal (CC) and mediolateral oblique (MLO) views were obtained, including digital breast tomosynthesis (Selenia Dimensions, Hologic Inc., Bedford, MA) and synthetic 2D images (C‐view, Hologic Inc., Bedford, MA).

The patient had an Abbott HeartMate 3 LVAD device which was placed approximately two years prior to the mammogram exam. LVAD settings were documented 6 weeks prior to the exam by a clinical engineer to be RPM 5000, Power 3.6 W, and Pulsatility 5.9 (summarized in Table 1).

**TABLE 1:** Summary of the patient's LVAD settings at the time of the affected mammographic exam/.

LVAD details	
Model	Abbott HeartMate 3
RPM	5000
Power	3.6 W
Pulsatility	5.9

## 3 RESULTS

**FIGURE 1:**
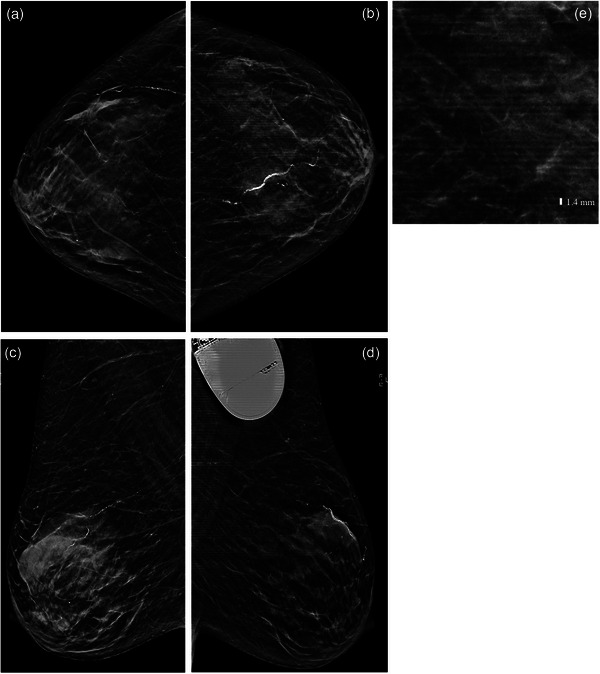
Screening mammogram of a patient with an LVAD, demonstrating periodic linear artifacts oriented in the anteroposterior dimension on left breast images but not the right. (a) Right CC view, (b) Left CC view, (c) Right MLO view, (d) Left MLO view, and (e) Close up of the outer left breast on CC view for zoomed‐in view of the observed artifact. Note the artifacts demonstrate a periodicity of ∼1.4 mm.

Linear artifacts oriented in the anteroposterior dimension were seen on all left breast images, whereas concurrent right breast images did not demonstrate any artifacts (Figure 1). These linear artifacts occurred in a spatially periodic pattern, with a periodicity of ∼1.4 mm (Figure 1e). Multiple repeat attempts were performed using two identical digital breast tomosynthesis units at our imaging center, all of which demonstrated the same artifacts. On the other hand, no other exam at our imaging center that day demonstrated any such artifacts, which excluded machine malfunction as the underlying etiology.

**FIGURE 2:**
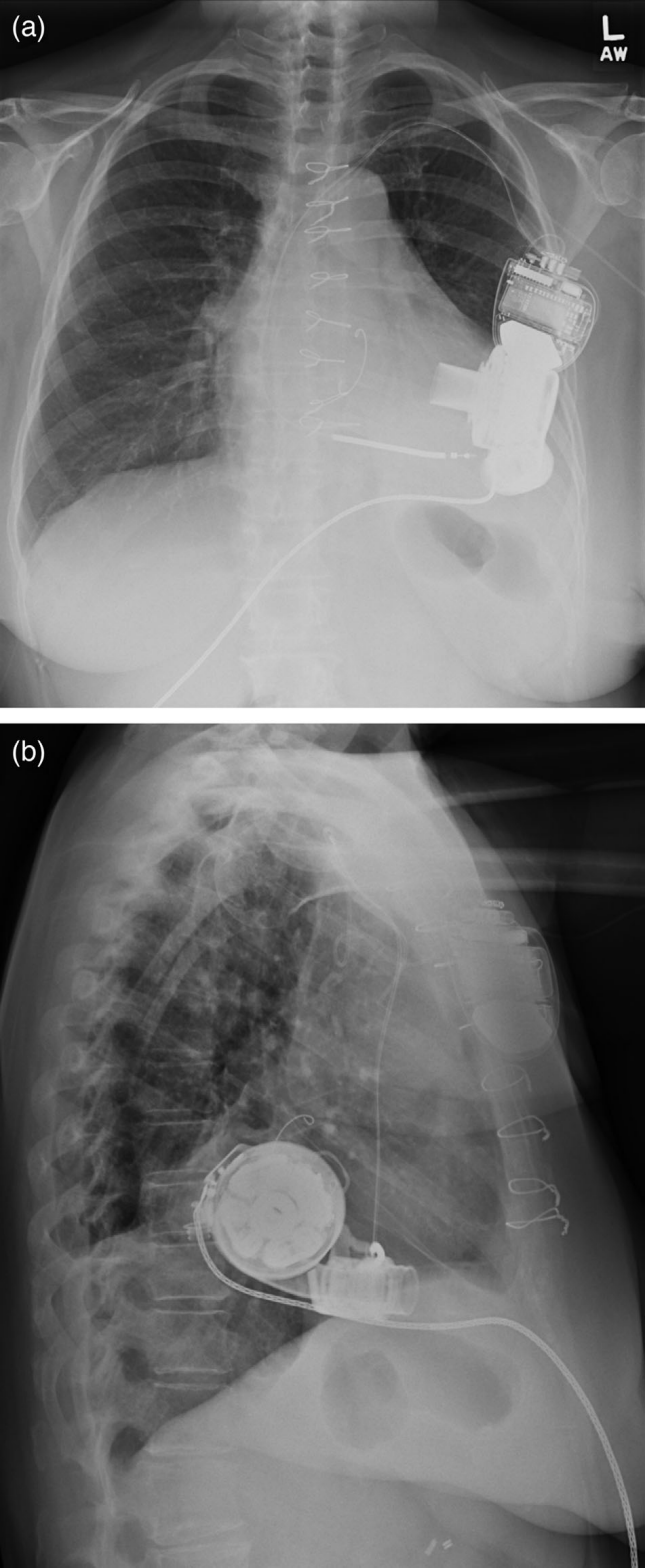
Chest radiograph exam demonstrating the relative location and proximity of the patient's LVAD to the left breast. Note this exam was obtained with computed radiography (CR) technology and not digital radiography (DR). In contrast to the patient's affected mammogram exam, LVAD related artifacts are absent on this chest radiograph. This is in keeping with a prior study by Wells et al.^19^ that demonstrated LVAD induced artifacts to affect only DR chest radiograph exams and not CR. (a) Frontal view. (b) Lateral view.

Of note, mammogram exams performed on this patient prior to her LVAD placement did not exhibit any similar artifacts. Also, LVAD location within the patient and proximity to the left breast, can be appreciated on a chest radiograph exam performed 10 months prior to the affected mammogram exam (Figure 2). It is also worth noting this chest radiograph exam did not exhibit any artifacts despite the presence of the LVAD and was acquired using computed radiography (CR) detector technology. A prior study by Wells et al. had shown that LVADs can cause similar linear artifacts on chest radiographs but only if acquired using digital radiography (DR) and not CR.^19^

## 4 DISCUSSION

As described above, we present a case of screening mammography with periodic linear artifacts likely due to the patient's HeartMate 3 LVAD. Presence of artifacts only in left breast images (and not in the right) supports the patient's LVAD as the underlying source, given the proximity of the LVAD to the left breast. The artifact pattern observed in our patient case is also similar to another previously reported case of LVAD induced artifact on mammography.^20^ Further, although the patient in our case also has a left chest wall automatic implantable cardioverter defibrillator (AICD), it is unlikely to be the artifact source, as patients with AICDs are routinely imaged without any mammographic artifacts. Prior studies have shown that AICDs can decrease mammographic image quality due to suboptimal patient positioning, decreased breast compression, and axillary tissue obscuration, but none have found AICDs to cause the artifacts seen in our patient case.^21^, ^22^

A recent study on chest radiographs in patients with LVADs by Wells et al. demonstrated similar linear artifacts.^19^ In that study, artifact prominence was shown to depend on distance from LVAD, which is in keeping with our observation of artifacts only in left breast images and not in the right breast.^19^ Furthermore, that study also demonstrated LVAD induced artifacts to be specific to digital radiography (DR) and absent on computed radiography (CR).^19^ This is also consistent with our observations, as the Hologic Selenia Dimensions machine utilizes DR technology. Further, our patient also received multiple chest radiograph exams acquired using CR technology after LVAD placement (Figure 2). In keeping with the findings of Wells et al, none of those CR chest radiographs demonstrated LVAD related artifacts.

Electromagnetic interference by LVADs has also been observed on electrocardiograms (ECGs) performed on LVAD patients.^23^ Thus, it is clear that LVADs can cause significant electronic interference on nearby devices. In that study, Loring et al. discussed methods to improve the affected ECGs using frequency filters.^23^ Similar approaches may also be of use in ameliorating LVAD artifacts on mammogram images, such as with the application of a notch/bandstop spatial frequency filter to affected images, although there may be tradeoffs with image blurring. Alternatively, a preferred approach may be to improve the electronic shielding of LVAD motors to reduce interference on nearby devices, and therefore prevent LVADs from causing artifacts on mammography. Active discussion by the imaging community with LVAD device manufacturers can help attract attention to this issue in future LVAD designs. Improvements in mammography room shielding may also be of value, particularly if the interference arises from external power sources, such as with the application of techniques discussed in a recent publication by Hintenlang et al.^24^ At the same time, for LVAD devices that are in close proximity to the detector, it may be more beneficial to consider shielding the detector while ensuring that it does not compromise image quality or affect radiation dose to the patient.

LVAD use has been steadily increasing worldwide,^18^ as well as survival rates of patients receiving LVADs.^16^ Current reported median survival after LVAD placement range up to 7 years,^25^, ^26^ and numerous patients are even living with LVAD devices for over 10 years.^27^, ^28^ Although there are no consensus guidelines, screening mammograms are typically considered worthwhile in patients with greater than 10‐year life expectancy.^29^, ^30^ Given the increasing survival rates, patients with LVADs may be undergoing screening mammography more routinely in the not‐too‐distant future. If so, it will be important to be aware of LVAD related artifacts in mammography. It may also be important to develop methods to reduce LVAD related mammographic artifacts, such as those suggested above.

## AUTHOR CONTRIBUTIONS

Jing‐Tzyh Alan Chiang initially identified the finding and developed the concept for the manuscript. Audrey I. Nisbet, David Ahmadian, Srinivasan Vedantham, and Jing‐Tzyh Alan Chiang contributed to the content of the manuscript, analysis of existing literature, compilation of references, and writing and editing of the manuscript. All authors contributed to the final manuscript and provided critical input.

## CONFLICT OF INTEREST STATEMENT

The authors declare no conflicts of interest.
